# Supplementary figures and images for: Epidemiological analysis of intramuscular hemorrhage of respiratory and accessory respiratory muscles in fatal drowning cases
Source: PLoS One. 2021 Dec 23;16(12):e0261348. doi: 10.1371/journal.pone.0261348 (PMC8699964; doi:10.1371/journal.pone.0261348)

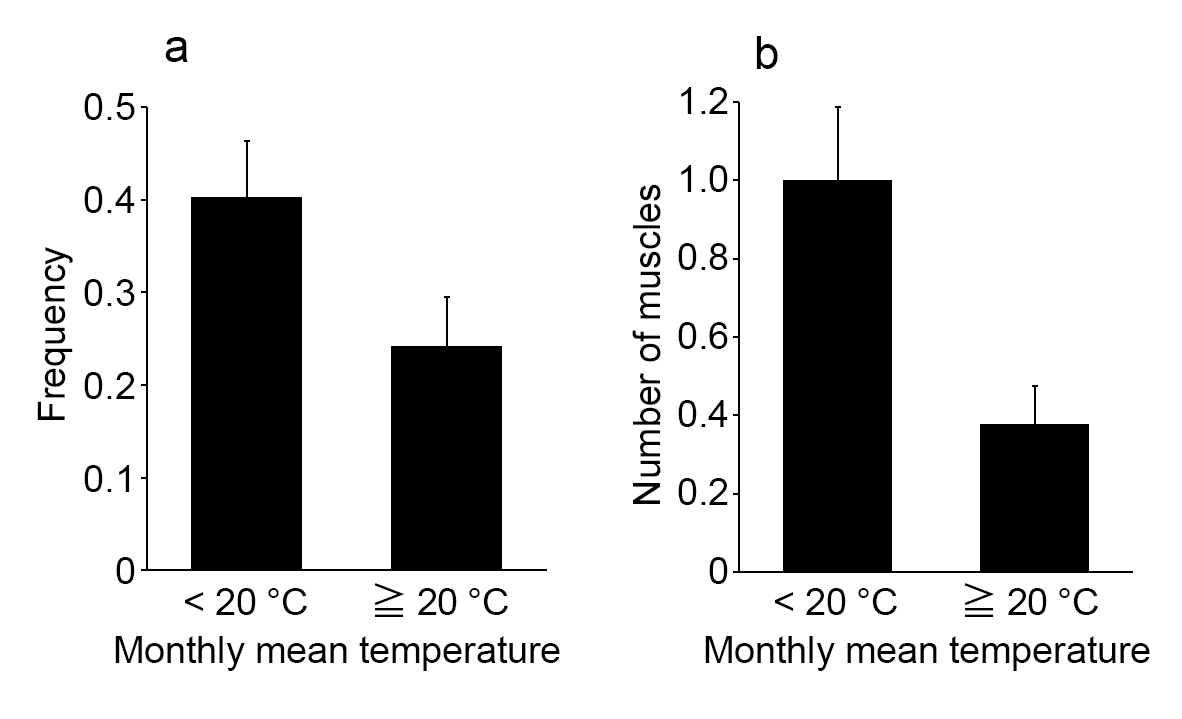

Supplement: S1 Fig — Comparison of intramuscular hemorrhage (a) and number of muscles with hemorrhage (b) between monthly mean temperature below 20°C and above 20°C, excluding cases of drowning in the bathtub. All values represent the mean ± SEM. (TIF) [file pone.0261348.s001.tif]

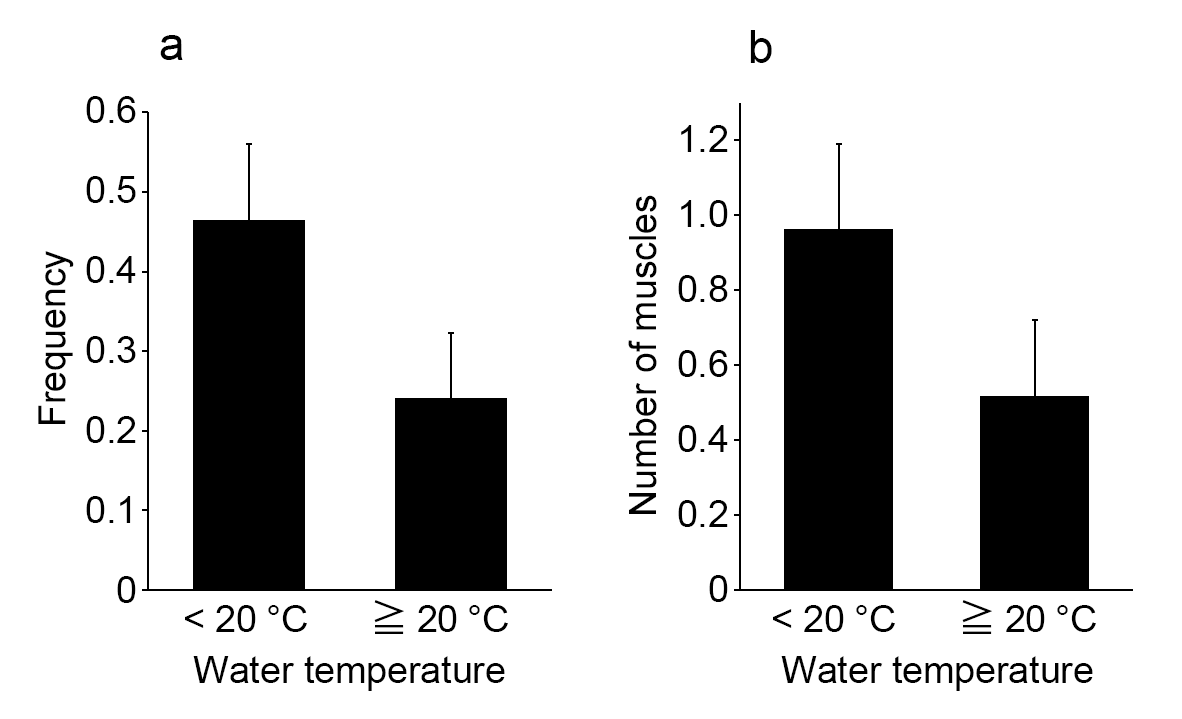

Supplement: S2 Fig — Comparison of the frequency of intramuscular hemorrhage (a) and the number of muscles with hemorrhage (b) between water temperature (<20°C and ≥20°C) at the time of discovery. All values represent the mean ± SEM. (TIF) [file pone.0261348.s002.tif]
