# Supplementary material for: Epidemiological analysis of intramuscular hemorrhage of respiratory and accessory respiratory muscles in fatal drowning cases
Source: PLoS One. 2021 Dec 23;16(12):e0261348. doi: 10.1371/journal.pone.0261348 (PMC8699964; doi:10.1371/journal.pone.0261348)
Supplement: Attachment — Submitted filename: Response to Reviewers.doc [file pone.0261348.s003.doc]

Dear Academic Editor and Reviewers,

We would like to thank you for your critical and instructive comments. We read the comments carefully and revised the manuscript in accordance with the reviewer’s comments, as follows. In the revised manuscripts, we indicate the modified points with highlights.

**Answers to Editor**

1. Following the editor's comment, we checked that our manuscript format conformed to the *PLOS ONE*’s format and corrected any non-conformities.

2. In accordance with the editor’s comment, we ensured that we have discussed whether all data/samples/autopsy reports were fully anonymized before we accessed them and/or whether the IRB or ethics committee waived the requirement for informed consent.

3. Following the editor's comment, I obtained an ORCID ID and clicked on the Fetch/Validate link next to the ORCID field in “Update my Information” page.

4. Figure 1 shows a representative selection of autopsy photographs taken by the authors during the autopsy, with information on the sex and age of the study sample added to the legend of Figure 1 (please see **page 6, lines136-138**). The use of autopsy photographs in the article has been approved by the Ethics Committee as long as no individual is identified. Therefore, the images are not copyrighted.

**Answers to Reviewer #1:**

Major comments

1. In accordance with your suggestion, we added discussion concerning the difference in intramuscular hemorrhage between freshwater and seawater drowning (please see **page 13, lines 284-289 of Discussion**).

2. We did not include cases from estuarine areas in our study sample because, as the reviewer stated, the mechanism of death in brackish water may be different from that in seawater or freshwater drowning. We are sorry for the omission in the original version of manuscript. We have added a note in the results section that cases in brackish water were excluded (please see **page 8, lines 174-175 of Result**).

Minor comments

1. In accordance with your suggestion, we set the month corresponding to every season (please see **page 9, lines 194-196 of Result**).

2. In accordance with your suggestion, we changed “in patients” to “in victims” in **new page 8, line 164**. We have also changed the legend in Figure 3 to reflect this (please see **legend to Fig 3, page 8, line 168**).

**Answers to Reviewer #2:**

1. In accordance with your suggestion, we have included in the Introduction a more detailed description of the morphological findings suggestive of vital drowning and have added a description of the difficulties in diagnosing drowning death (please see **pages 3-4, lines 46-76 of Introduction**). We have also these contents to the Abstract, accordingly (please see **page 2, line 23-25 of Abstract**).

2. In reply to your suggestion, we noted that the usefulness of the plankton test is also debatable (please see **page 4, lines 77-82 of Introduction**).

3. Since we also believe that the possibility of blunt external force as a cause of the intramuscular hemorrhage assessed in this study should be ruled out, we have added descriptions in the Abstract (please **see page 2, lines 38-41**), Discussion (please see **page 12, lines 263-266**), and Conclusion sections (please see **page 15, lines 333-336**), respectively. We also added that intramuscular hemorrhage is also observed in other causes of death than drowning (please see **page 12, lines 261-263**).

4. In accordance with your suggestion, as it is inappropriate to estimate the elapsed time of drowning on the presence of fluid blood or blood with soft clots, we have deleted and modified those statements (please see **page 14, lines 297-301**). We have also deleted Suppl. Fig 2, accordingly.

5. In accordance with your suggestion, we examined the correlation between temperature and age and found no significant difference (please see **page 5, lines 111-112 of Materials and methods, page 10, lines 214-215 of Results**). Therefore, it is stated that low temperature and age are independent factors that predispose to intramuscular hemorrhage (please see **page 2, lines 41-43 of Abstract, page 14, lines 310-311 of Discussion**).

6. In accordance with your suggestion, we investigated the relationship between the occurrence of intramuscular hemorrhage and a potential bleeding tendency caused by disease (liver cirrhosis) and drugs (anticoagulants). The results showed that both the frequency of intramuscular hemorrhages and the number of muscles with hemorrhage were not affected by the presence or absence of bleeding tendency. This is a useful result and we have added a description of this to the Abstract (please see **page 2, lines 36-38**), Materials and methods (please see **page 5, lines 100-101**), Results (please see **page 11, lines 231-236**), new Fig 7 with legend (please see **page 11, lines 238-240**), and Discussion (please see **page 15, lines 323-329, page 16, lines 340-341**).

7. In reply to your suggestion, we modified the phrases in our text in a logical order (please see **page 8, lines 163 and 165, page 10, lines 212 and 214, page 11, lines 225 and 226**).

8. In accordance with your suggestion, we have revised the first draft because the conclusions were overstated. We have kept these intramuscular hemorrhages to the extent that they give us a clue to differentiate between drowning death and other causes of death, if we can completely rule out the possibility of trauma or other causes of death (please see **page 2, lines 38-41, page 15, lines 333-336**). We deleted the mention of age estimation.

We hope that the revised manuscript is found to be much improved and suitable for the publication in *PLOS ONE*. We thank you in advance for your great kindness and look forward to your favorable reply.

With my best regards,
